# Heat-Not-Burn cigarette induces oxidative stress response in primary rat alveolar epithelial cells

**DOI:** 10.1371/journal.pone.0242789

**Published:** 2020-11-25

**Authors:** Yoko Ito, Kana Oshinden, Naokata Kutsuzawa, Chinatsu Kohno, Sanae Isaki, Keiko Yokoyama, Tadayuki Sato, Masayuki Tanaka, Koichiro Asano

**Affiliations:** 1 Division of Pulmonary Medicine, Department of Medicine, Tokai University School of Medicine, Isehara, Kanagawa, Japan; 2 Department of Bioinformatics and Molecular Biology, Support Center for Medical Research and Education, Tokai University School of Medicine, Isehara, Kanagawa, Japan; University of Alabama at Birmingham, UNITED STATES

## Abstract

There has been an increase in the usage of heat-not-burn (HNB) cigarette products. However, their effects on alveolar epithelial cells (AECs) remain unknown. AECs are the target cells of conventional cigarette smoking-related respiratory diseases such as chronic obstructive pulmonary disease, idiopathic pulmonary fibrosis and lung cancer whose pathogenesis involves oxidative stress. In this study, primary rat AECs were isolated, cultured and stimulated by HNB cigarette smoke extract (CSE). Our data indicate that rat AECs exposed to HNB CSE induced oxidative stress response genes (e.g. *Hmox-1*, *Gsta1*, *Gsta3 and Nqo1*). We also compared the oxidative stress response between two different types of AECs, alveolar type I-like (ATI-like) cells and type II (ATII) cells, and between two different types of cigarette, HNB cigarettes and conventional cigarettes. The expressions of *Gsta1*, *Gsta3* and *Nqo1* were higher in ATII cells than ATI-like cells in response to HNB and conventional cigarettes, but there was no significant difference in their expression levels between HNB cigarette and conventional cigarette. Taken together, our results suggest that HNB cigarettes have the similar potential as conventional cigarette products to induce oxidative stress response in AECs.

## Introduction

Cigarette smoking is well-known to cause disastrous respiratory diseases such as chronic obstructive pulmonary disease (COPD), idiopathic pulmonary fibrosis (IPF) and lung cancer [1]. Newer forms of smoking devices, known as electric cigarettes (e-cigarettes) and heat-not burn (HNB) cigarettes, have increased in popularity in the past decade. E-cigarettes with nicotine are not publicly sold in Japan except for personal import, as they are regulated by the Medical Products and Medical Devices Act under the jurisdiction of the Ministry of Health, Labor and Welfare. Meanwhile, in 2016 Japan became the first country in the world to sell I-Quit-Ordinary-Smoking (IQOS), one of HNB cigarette products. The tobacco company selling IQOS claimed it to be safer and less harmful products because the levels of some toxicant are lower in IQOS than in conventional cigarettes, although the IQOS aerosol still contains significant levels of toxicants known as volatile organic compounds including acrolein, acetaldehyde and formaldehyde [2]. Consequently, IQOS had 98% of the world market share in 2016 [3] and even now it has about 70% of the domestic share among HNB cigarette products in Japan.

AECs play critical roles in the pathogenesis of cigarette smoke-related respiratory diseases. The alveolar epithelium consists of two cell types, type I alveolar epithelial (ATI) cells and type II alveolar epithelial (ATII) cells. ATI cells are responsible for gas exchange, and liquid homeostasis in the alveoli, but are highly susceptible to injury and incapable of self-renewal. In contrast, ATII cells are highly resistant to insults, able to self-renew, and repopulate ATI cells [4].

Since HNB cigarettes are still relatively new products, it is imperative to confirm whether HNB cigarettes are harmful to AECs before users get diagnosed with HNB cigarette-induced respiratory diseases. Here, we examine the effects of cigarette smoke extract (CSE) from HNB cigarettes on primary rat AECs.

## Materials and methods

### Rat ATII cell isolation and culture

ATII cells were isolated from pathogen-free, adult, male Sprague Dawley rats (weighing 240–350 g; Charles River Laboratories Japan, Inc., Yokohama, Kanagawa, Japan) which were euthanized with nembutal (100 mg/ body) by dissociation with porcine pancreatic elastase (Worthington, Lakewood, NJ., USA) and partial purification on discontinuous density gradients, according to the methods described previously [5]. The freshly isolated cells in 10% dimethyl sulfoxide (DMSO) and 90% fetal bovine serum (FBS) had been frozen in nitrogen liquid until further use. This research was approved by the Animal Care Committee at Tokai University School of Medicine.

For culture, frozen rat ATII cells were resuspended in Dulbecco’s minimal essential medium (DMEM). Next, to transdifferentiate ATII cells into ATI-like cells, ATII cells were cultured on rat tail collagen I (RTC; Gibco Thermo Fischer Scientific, Waltham, MA., USA)-coated tissue culture plates. Cells were plated in DMEM with 10% FBS for one day and subsequently cultured in DMEM with 5% FBS for 2 days.

To maintain ATII cells in differentiated state, they were plated on millicell inserts (MilliporeSigma, Burlington, MA., USA), that had been previously coated with a mixture of 20% Engelbreth-Holm-Swarm tumor matrix (Corning, Corning, NY., USA) and 80% RTC in DMEM with 10% FBS, for one day and then cultured for two days with 5% FBS along with 10 ng/ml human keratinocyte growth factor (R&D Systems Inc., Minneapolis, MN, USA).

### Preparation of CSE

HNB CSE was generated using one IQOS heat-stick (Philip Morris Japan, Tokyo, Japan) heated for 6 minutes (min) because the heating duration of IQOS 3 holder is limited to 6 min. Corresponding conventional CSE was generated using one Marlboro Red cigarette (Philip Morris Japan, Tokyo, Japan) (1.0 mg nicotine) until it burned completely. The IQOS aerosol and the Marlboro Red cigarette smoke were individually bubbled through a 25 mL tissue culture flask containing 12.5 mL serum-free DMEM at a constant rate with a modification of Health Canada Intense regime (taking one puff of 55 mL volume for 2 seconds (sec) every 30 sec with a 100% blockade of the ventilation zone on the cigarette filter) [6]. The freshly generated CSEs from IQOS and Marlboro Red (100%) were filtered through a 0.22 μm pore-sized PVDF syringe filter and immediately applied to the cell cultures.

### Lactate dehydrogenase (LDH) assay

To examine cytotoxicity levels in ATI-like cells exposed to IQOS CSE for 6 and 24 hours (h), LDH levels in the culture medium were measured using the cytotoxicity LDH assay kit (Dojindo Molecular Technology, Inc., Kumamoto, Japan) according to the manufacturer’s instruction.

### Microarray experiments

At 6 and 24 h after IQOS CSE stimulation, total RNA from ATI-like cells was extracted and purified using RNeasy kit (Qiagen, Hilden, Germany). The samples were run on Agilent SurePrint G3 (Agilent, Santa Clara, CA, USA) and processed as indicated by the manufacturer. Raw signal values were normalized using the 75th percentile and transformed log2 scale. The processed data was analyzed using Genespring GX version 14.9 (Agilent).

### Immunocytochemistry

To detect NRF2 translocation induced by IQOS CSE, ATI-like cells cultured on a 12 well hanging insert (control cells and cells exposed to 20% IQOS CSE for 0.5, 1, 2 and 4 h) were fixed in 4% paraformaldehyde. After blocking with 3% normal goat serum (Rockland Immunochemicals, Inc., Limerick, PA, USA) in PBS, the cells were incubated with rabbit anti-NRF2 (N2C2) antibody (GeneTex, Inc., Irvine, CA, USA) overnight. Next, cells were incubated with the secondary antibody, Alexa Fluor 594 goat anti-rabbit IgG (Invitrogen, Carlsbad, CA, USA) for 1 h, and subsequently, the cells were mounted with Vectashield medium containing DAPI (Vector laboratories, Burlingame, CA, USA).

### Real-time RT-PCR

For real-time RT-PCR, the expression levels of genes were expressed as a ratio to the expression of the constitutive probe peptidylprolyl isomerase A *(Ppia)*. The specific primers and probes for Heme Oxygenase-1 *(Hmox-1)*, Glutathione S-Transferase Alpha 1 *(Gsta1)*, Glutathione S-Transferase Alpha 3 *(Gsta3)*, *and* NAD(P)H Quinone Dehydrogenase 1 *(Nqo1)* were purchased from Applied Biosystems (Waltham, MA., USA).

### Western blotting

Protein expression in ATI-like cells exposed to IQOS CSE for 24 h was measured by Simple Western System Wes (ProteinSimple, Inc., San Jose, CA, USA) according to the manufacturer’s instruction. Protein loading was normalized to rabbit anti-glyceraldehyde-3-phosphate dehydrogenase (anti-GAPDH) purchased from Sigma-Aldrich (St. Louis, MO, USA). Mouse anti-HMOX-1 antibody and rabbit anti-NQO1 antibodies were purchased from Abcam (Cambridge, UK) and GeneTex, Inc., respectively, and anti-GSTA1 and GSTA3 were purchased from proteintech (Rosemont, IL, USA).Goat anti-rabbit secondary horseradish peroxidase (HRP) antibody and goat anti-mouse secondary HRP antibody were purchased from ProteinSimple, Inc. Images were quantitated using the Compass software (ProteinSimple, Inc.).

### Statistical analysis

One-way ANOVA was performed using GraphPad Prism 7.04 to evaluate statistical differences among experimental groups. Tukey’s post hoc test was applied for multiple comparisons and results with p < 0.05 were considered statistically significant. All data are shown as the mean ± standard error of the mean (SEM) from three independent experiments.

## Results

### Cytotoxicity was not detected in ATI-like cells at 24 h after IQOS CSE exposure

The LDH assay was used to evaluate the effect of IQOS CSE on cytotoxicity in rat ATI-like cells. The cytotoxicity was induced in ATI-like cells only by 20% IQOS CSE at 6 h, but not in ATII cells by either condition (S1A Fig). At 24h the cytotoxicity was not induced by either 10, 20% IQOS or 10% Marlboro Red CSE (S1B Fig).

### Top ten genes upregulated by 20% IQOS CSE in ATI-like cells by microarray analysis

To explore which genes in ATI-like cells were modified by IQOS CSE, a microarray analysis was conducted with samples from cells stimulated by 10 or 20% IQOS CSE for 6 h. Two oxidative stress response genes, *Gsta3* and *Hmox-1*, were identified among the top ten genes upregulated by 20% IQOS CSE at 6 h (Table 1). As oxidative stress plays pivotal roles in the pathogenesis of various respiratory diseases, especially common respiratory diseases like COPD, IPF and lung cancer, we decided to focus on the oxidative stress response genes in this study.

**Table 1:** Top ten genes upregulated by 20% IQOS CSE at 6 h after exposure.

Gene Symbol	Description	Fold Change 10% IQOS vs Control	Fold Change 20% IQOS vs Control
*Lhx5*	Rattus norvegicus LIM homeobox 5 (Lhx5), mRNA [NM_139036]	3.42	221.57
***Gsta3***	Rattus norvegicus glutathione S-transferase alpha 3 (Gsta3), transcript variant 1, mRNA [NM_001009920]	3.19	10.67
*ZSCAN29*	zinc finger and SCAN domain containing 29 [Source:RGD Symbol;Acc:1559150] [ENSRNOT00000035889]	7.24	8.13
***Hmox1***	Rattus norvegicus heme oxygenase 1 (Hmox1), mRNA [NM_012580]	3.01	7.89
*RGD1563231*	similar to immunoglobulin kappa-chain VK-1 [Source:RGD Symbol;Acc:1563231] [ENSRNOT00000074382]	7.09	7.19
*Itgb3*	Rattus norvegicus integrin subunit beta 3 (Itgb3), mRNA [NM_153720]	4.74	5.13
*Glt1d1*	glycosyltransferase 1 domain containing 1 [Source:RGD Symbol;Acc:1304896] [ENSRNOT00000064526]	6.05	5.10
*Mest*	Rattus norvegicus mesoderm specific transcript (Mest), mRNA [NM_001009617]	3.25	4.75
*Prl8a7*	Rattus norvegicus prolactin family 8, subfamily a, member 7 (Prl8a7), mRNA [NM_022537]	3.56	4.05
*Best3*	Rattus norvegicus bestrophin 3 (Best3), mRNA [NM_001191783]	4.02	3.83

### IQOS CSE induced nuclear localization of NRF2 in ATI-like cells at 2 h after stimulation

**Fig 1:**
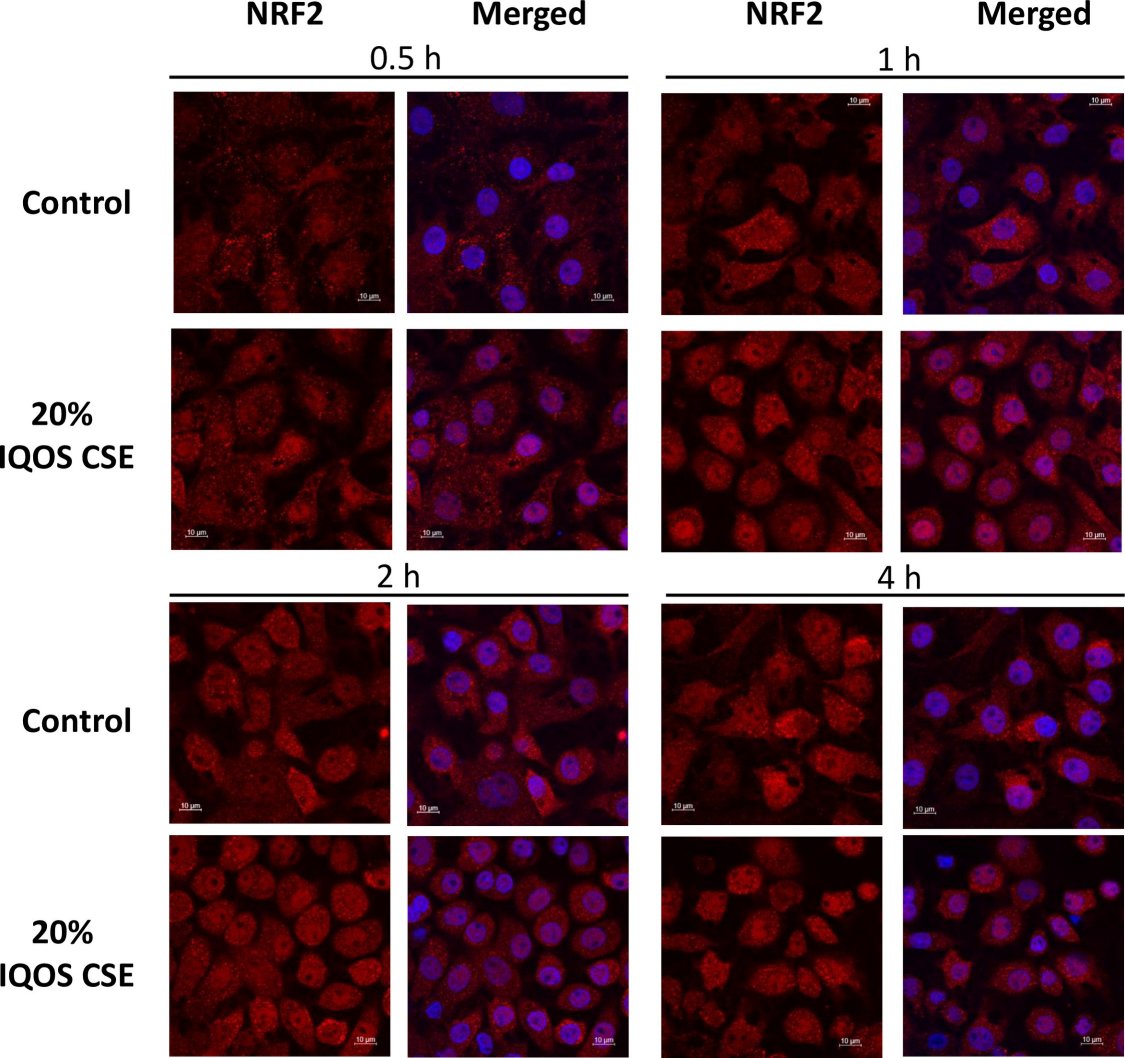
Nuclear translocalization of NRF2 in rat ATI-like cells is detected using immunocytofluorescence at 0.5, 1, 2 and 4 h after stimulation with 20% IQOS CSE. NRF2 (red), DAPI (blue).

NRF2 is a transcription factor, which plays an important role in the cellular defense against oxidative stress [7]. In response to oxidative stress, NRF2 rapidly translocates to the nucleus and induces mRNA expression of various antioxidant genes, such as *Hmox-1*, *Gsta1*, *Gsta3* and *Nqo1*. Stimulation by IQOS CSE (20%) induced translocalization of NRF2 protein from cytoplasm to nuclei in ATI-like cells starting from at 0.5 h (Fig 1).

### The expression of NFR2 target genes in ATI-like cells was upregulated by IQOS CSE exposure

**Fig 2:**
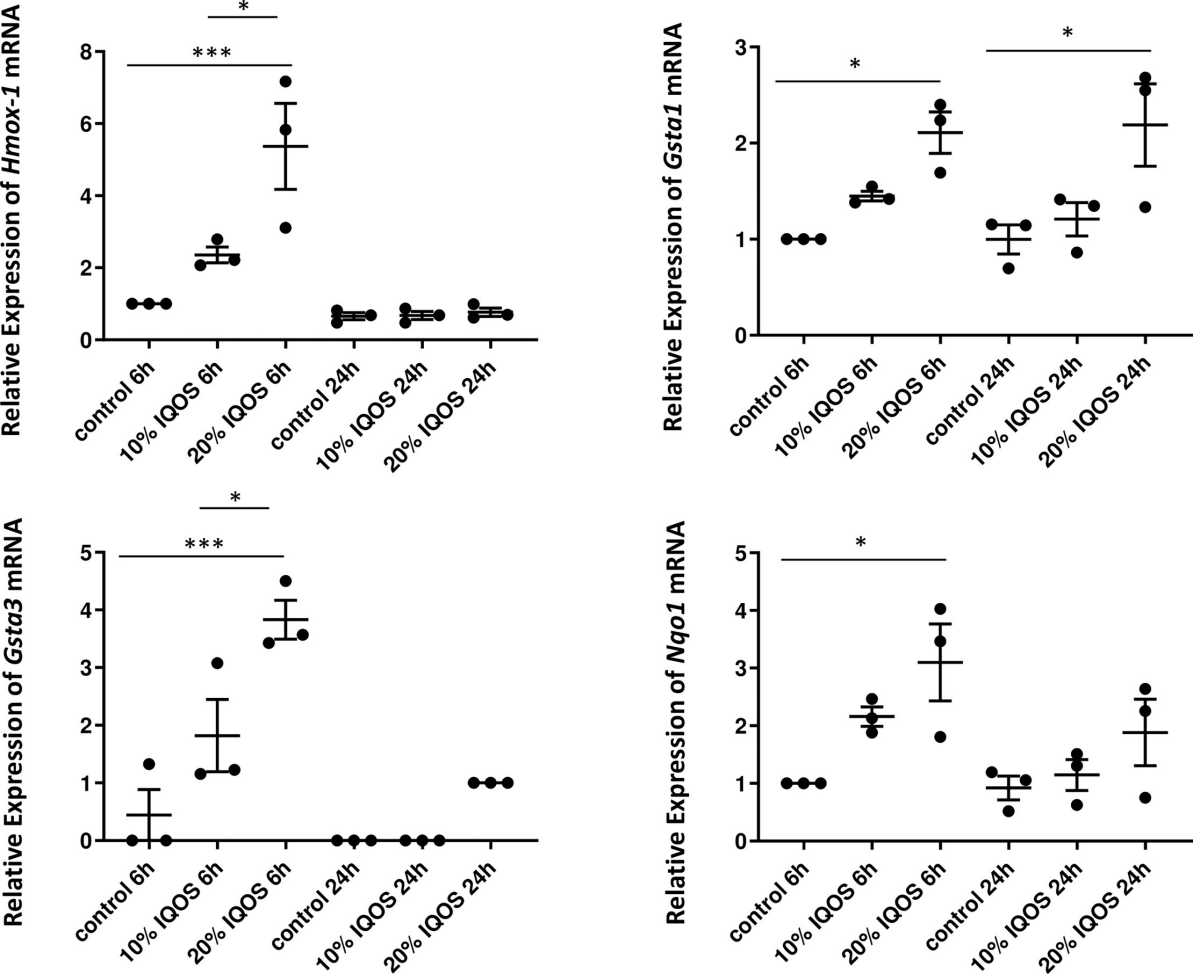
The expression levels of *Hmox-1*, *Gsta1*, *Gsta3* and *Nqo1* mRNA in rat ATI-like cells are upregulated at 6 h after stimulation with 10% and/or 20% IQOS CSE. Only *Gsta1* mRNA expression is upregulated at 24 h after stimulation with 20% IQOS CSE. Data represent results from three independent experiments. Each experiment was done in duplicate or triplicate wells. *: p < 0.05, ***: p < 0.001.

**Fig 3:**
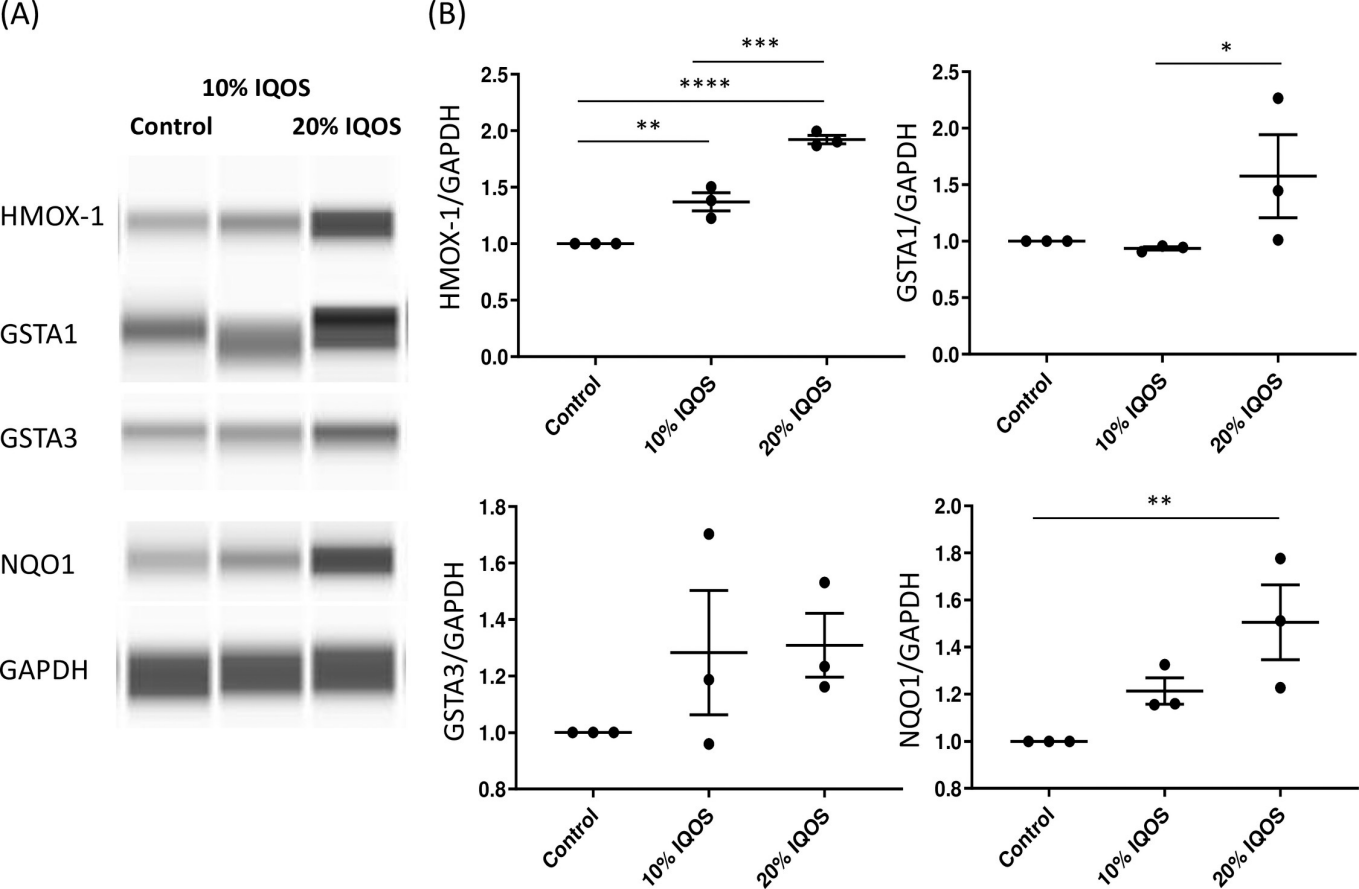
HMOX-1 and NQO1 but not GSTA1 and GSTA3 proteins in rat ATI-like cells are induced at 48 h after 10% and/or 20% IQOS CSE stimulation. A) representative blot, B) quantitation of HMOX-1, GSTA1, GSTA3 and NQO1 relative protein expression. Data represent results from three independent experiments. *: p < 0.05, **: p < 0.01, ***: p < 0.001.

To confirm the upregulation of NRF2 target genes in ATI-like cells, the mRNA expression levels of *Hmox-1*, *Gsta1*, *Gsta3* and *Nqo1* were measured by quantitative real time RT-PCR at 6 and 24 h after 10 or 20% IQOS CSE exposure (Fig 2). All four genes were induced at 6 h after IQOS CSE stimulation. The mRNA expression levels of *Hmox-1* and *Gsta3* which were included in top ten genes by microarray analysis were upregulated in a dose-dependent manner. However, at 24 h, only *Gsta1* mRNA levels were statistically significantly upregulated. Next, the protein levels of HMOX-1, GSTA1, GSTA3 and NQO1 in ATI-like cells were investigated using western blot assay. The protein levels of HMOX-1 and NQO1 were increased at 24 h in a dose-dependent manner (Fig 3). However, the protein levels of GSTA1 and GSTA3 were not statistically upregulated at 24 h.

### The mRNA expressions of oxidative stress response genes except for Hmox-1 were higher in ATII cells than ATI-like cells

As ATI cells are known to be more vulnerable to injury than ATII cells, the difference in the induction of oxidative stress response genes was examined.

**Fig 4:**
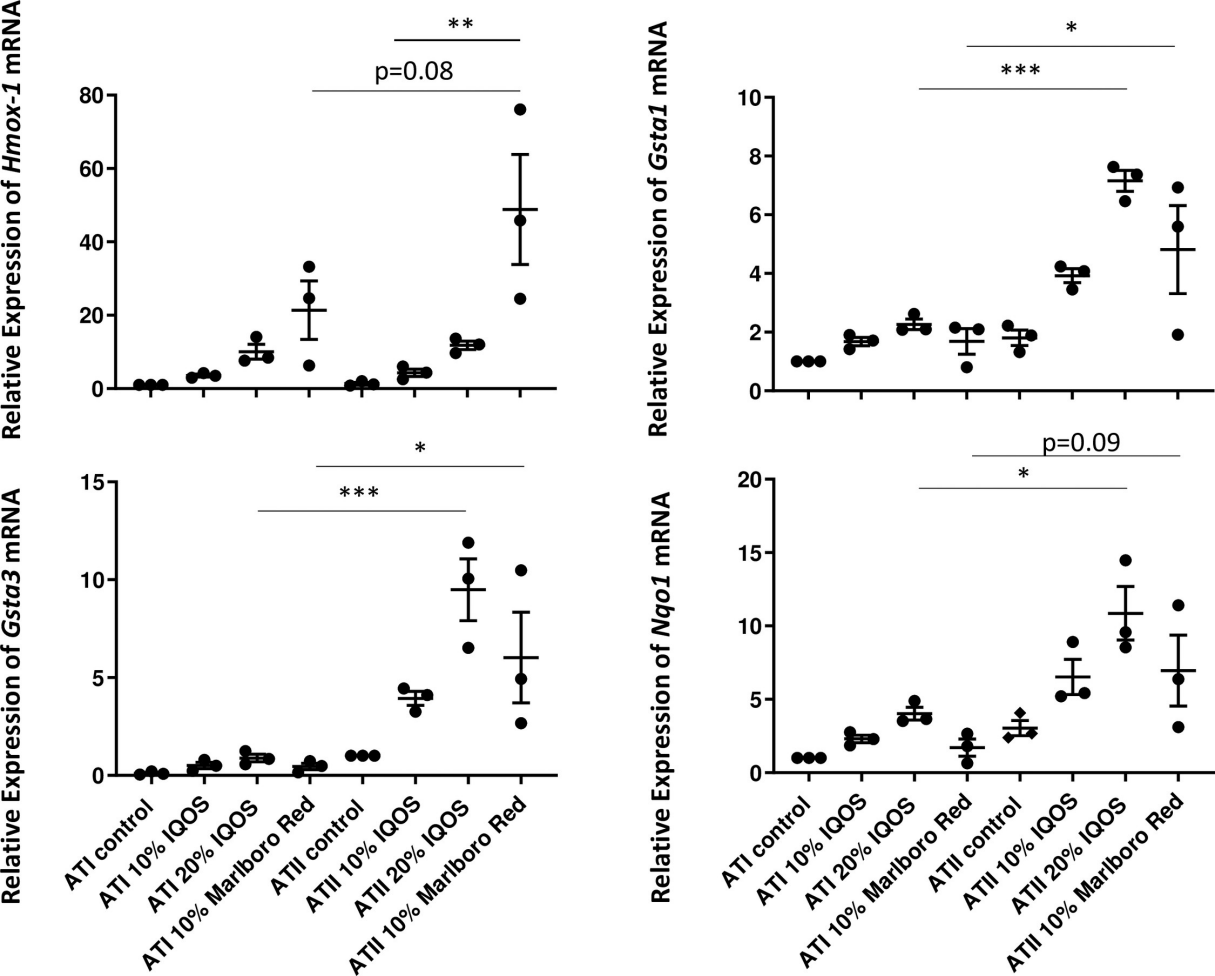
The comparison of *Hmox-1*, *Gsta1*, *Gsta3* and *Nqo1* mRNA expression between rat ATI-like cells and ATII cells exposed to CSE, and the comparison of these mRNA expression levels between IQOS CSE and Marlboro Red CSE. Data represent results from three independent experiments. Each experiment was done in duplicate or triplicate wells. *: p < 0.05, **: p < 0.01, ***: p < 0.001.

ATII cells showed higher expression levels of *Gsta1*, *Gsta3* and *Nqo1* mRNA levels upon 20% IQOS CSE or 10% Marlboro Red CSE stimulation when compared to ATI-like cells (Fig 4). However, no difference was observed in *Hmox-1* mRNA levels between the two different types of cells under any condition.

### There was no difference in those expressions between IQOS and Marlboro Red CSE exposure to both types of cells

To compare the effect of HNB and ordinary cigarette products on AECs, cells were stimulated by IQOS CSE and the CSE from the corresponding conventional cigarette product, Marlboro Red. The expression levels of *Gsta1*, *Gsta3* and *Nqo1* mRNA upon 10% IQOS CSE and 10% Marlboro Red CSE exposure were comparable in ATI-like and ATII cells and no statistical difference was seen (Fig 4). On the contrary, *Hmox-1* mRNA expression level was much higher upon 10% Marlboro Red CSE than 10% IQOS CSE exposure to ATII cells.

## Discussion

Despite claims of reduced harmful effects by the tobacco industry, adverse health effects of HNB cigarettes on the AECs have not been completely understood. Our study indicates that IQOS induces oxidative stress response in rat AECs, which suggests that HNB cigarettes have the potential to induce oxidative stress in the airways and cause development of oxidative stress-related respiratory diseases. To the best of our knowledge, this is the first report to show the effect of acute HNB CSE exposure on AECs which are the target cells for respiratory diseases.

NRF2, a master antioxidant transcription factor, is expressed abundantly in the lungs and plays protective roles, including maintenance of cellular redox homeostasis and reduction of severe oxidative damage, to prevent the development of the lung diseases [1]. Our results indicate that IQOS CSE activates NRF2 and upregulates the expression levels of oxidative stress response genes, such as *Hmox-1*, *Gsta1*, *Gsta3* and *Nqo1*, which is consistent with the previous studies on conventional cigarette products [8, 9]. Indeed, it has already been shown that gas phase radicals are detected in aerosol from both IQOS and conventional cigarette products, although particulate-phase radicals are not detected in IQOS [10]. As oxidative stress is involved in the occurrence and development of various respiratory diseases including COPD, IPF and lung cancer [1], our data indicate that HNB cigarettes can lead to these diseases by inducing oxidative stress in AECs.

Although cytotoxicity on ATI-like cells by IQOS CSE was seen at 6 h, our study does not detect cytotoxic effects of IQOS CSE on ATI-like cells with LDH assay at 24 h. Interestingly, this result supports recent studies which reported that IQOS aerosol exposure of various types of cells from respiratory system does not decrease cell viability through leakage of plasma membrane with LDH assay; however it affects critical cell functions such as metabolic activity via mitochondrial reductase function, as seen with MTT assay and lysosomal activity with neutral red uptake (NRU) assay [11, 12]. A possible reason for IQOS CSE not inducing cytotoxicity measured by LDH assay at 24 h might be that our experiment is performed with a single IQOS stick exposure.

When compared to Marlboro Red CSE, IQOS CSE exposure does not show any differences in the induction levels of *Gsta1*, *Gsta3* and *Nqo1* in both ATI-like cells and ATII cells. Previous *in vitro* study using human airway epithelial and smooth muscle cells found that IQOS aerosol and conventional cigarette smoke have a similar potential to increase oxidative stress, inflammation, airway remodeling and extracellular acidification rate [13]. An *in vivo* rat experiment showed that serum nicotine levels in IQOS-exposed rats are over four times higher than in conventional cigarette-exposed rats; further exposure to IQOS aerosol causes same extent of endothelial dysfunction in rats as exposure to conventional cigarettes [14]. Even data from a tobacco company showed that IQOS inhalation causes significant epithelial hyperplasia and metaplasia compared to controls in rat models, though to a lesser extent than what is observed upon conventional cigarette exposure [15]. Moreover, IQOS-exposed rats develop higher systemic neutrophilia and higher levels of thymic atrophy than control and conventional cigarette-exposed rats [15]. Recent human studies conducted on IQOS users in Japan and USA also revealed that there is no statistically detectable difference between IQOS and conventional cigarette users for biomarkers of potential harm such as inflammation, oxidative stress, cholesterol and triglycerides, blood pressure and lung function [16, 17]. Therefore, our results support these previous studies and suggest that HNB cigarettes induce oxidative stress levels in AECs similar to conventional cigarettes.

On the contrary, we have found that *Hmox-1* expression is higher in Marlboro Red-exposed AECs than in IQOS-exposed cells. In contrast to the indirect antioxidant enzymes referred as phase 2 detoxifying enzymes such as GSTA1, GSTA3 and NQO1 [1], HMOX-1 is considered as a stress response protein because it is regulated by diverse protein phosphorylation-dependent signaling pathways upon activation of a wide variety of transcription factors such as NRF2, MAPK, BTB (Bric a brac, Tramtrack and Broad complex), BACH1 (cap ‘n’ collar (CNC) homologue 1), AP-1 and NF-κB [18]. Therefore, based on our results, we speculate that IQOS might induce oxidative stress at similar levels as conventional cigarette exposure, but induction of other stresses might be higher with conventional cigarettes than with IQOS. However, this does not necessarily translate into lower stress and harm when people use HNB cigarettes compared to conventional cigarettes. Like the fact that passive smokers who breathe in much less toxic smoke than active smokers can also develop heart and lung diseases, even low levels of exposure to some toxins can be harmful [19]. In addition, even though IQOS heat stick is not set on fire, it is heated to 350°C, which is still hot enough to cause pyrolysis. Moreover, there is the evidence that smoking duration alone provides stronger risk estimates of COPD than pack-years [20]. Therefore, HNB cigarettes should not be marketed as safer and harm reduction products to human health.

Since rat ATI cells have not been isolated and cultured, we used ATI-like cells, generated from transdifferentiation of cultured ATII cells *in vitro*. The gene expression is quite similar to ATI cells, with some differences [21]. Consistent with previous studies that showed the different responses to viral infection and conventional cigarettes between ATI-like cells and ATII cells [8, 22], we have found that in contrast to ATI-like cells ATII cells does not show cell cytotoxicity by IQOS CSE at 6 h and that ATII cells express and induce more oxidative stress response genes than ATI-like cells in response to IQOS CSE. ATI cells are large flat cells and cover about 95% of the alveolar surface area that is directly exposed to the external environment, while ATII cells are cuboidal cells and cover only 5% of the alveolar surface. In addition, ATII cells are the progenitor cells for the alveolar epithelium and are responsible for reforming the alveolar epithelium after damage to ATI cells and they are thought as the defenders of the alveolar microenvironment [23]. Considering these differences in the cell characteristic between ATI and ATII cells, ATII cells should have stronger defense system against external stimuli than ATI cells, producing and releasing more oxidative stress response proteins.

Although our data suggest that HNB cigarettes are not harmless to the lungs, there are several limitations to this study. First, we observed only acute response to IQOS CSE generated from one IQOS stick. Second, we used an *in vitro* system with cells from rats, not humans. Thus, long term, repeated exposure experiments and *in vivo* studies with animals and/or HNB cigarette users must be conducted to verify our *in vitro* cell-based results. Nevertheless, our acute exposure model is reliable enough to conclude that HNB cigarettes have the potential to cause lung injury since our data are not contradictory to several previous studies [11–14, 16, 17]. Furthermore, two case reports which showed HNB cigarettes cause acute eosinophilic pneumonia, also strongly support that HNB cigarette induces damage to the lungs [24, 25].

In conclusion, this study has shown that HNB CSE induces oxidative stress response in rat AECs. Our results highlight the possibility that present HNB cigarette users may suffer from chronic lung diseases such as COPD, IPF, and lung cancer in future. Accumulation of scientific evidence about HNB cigarette-induced health problems is urgently needed for HNB cigarette users to prevent repetition of the history of health damage in conventional cigarette users.

## Supporting information

S1 Fig Cytotoxicity is detected by LDH assay only in rat ATI-like cells exposed to 20% IQOS CSE at 6 h (A). However, cytotoxicity is not detected in rat ATI-like cells exposed to 10% and 20% IQOS CSE and 10% Marlboro Red (MR) CSE at 24 h. Data represent results from three independent experiments. Each experiment was done in triplicate wells.
(TIF)

S2 Fig (JPG)

S3 Fig (JPG)

S4 Fig (JPG)

S5 Fig (JPG)

S6 Fig (JPG)
