# Supplementary figures and images for: Heat-Not-Burn cigarette induces oxidative stress response in primary rat alveolar epithelial cells
Source: PLoS One. 2020 Nov 25;15(11):e0242789. doi: 10.1371/journal.pone.0242789 (PMC7688177; doi:10.1371/journal.pone.0242789)

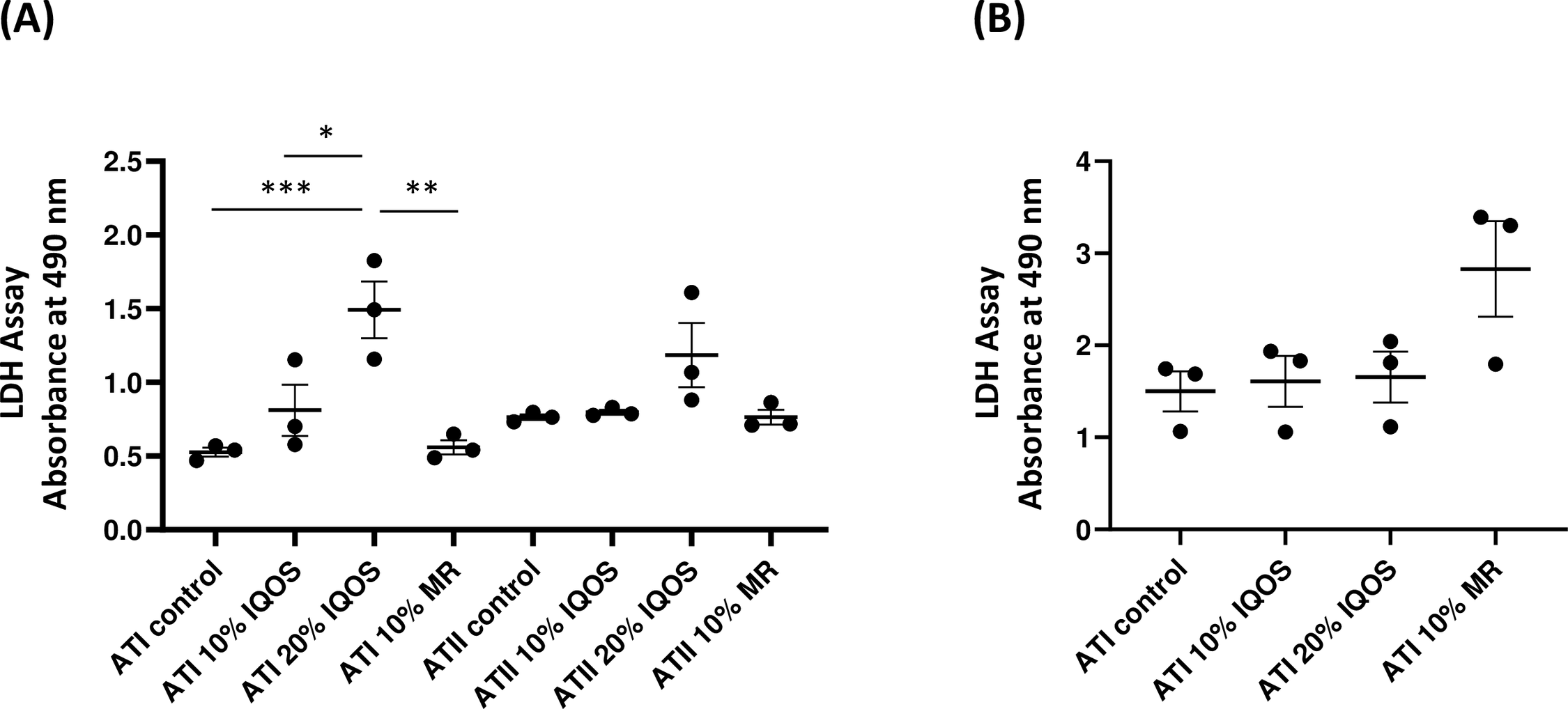

Supplement: S1 Fig — Cytotoxicity is detected by LDH assay only in rat ATI-like cells exposed to 20% IQOS CSE at 6 h (A). However, cytotoxicity is not detected in rat ATI-like cells exposed to 10% and 20% IQOS CSE and 10% Marlboro Red (MR) CSE at 24 h. Data represent results from three independent experiments. Each experiment was done in triplicate wells. (TIF) [file pone.0242789.s001.tif]

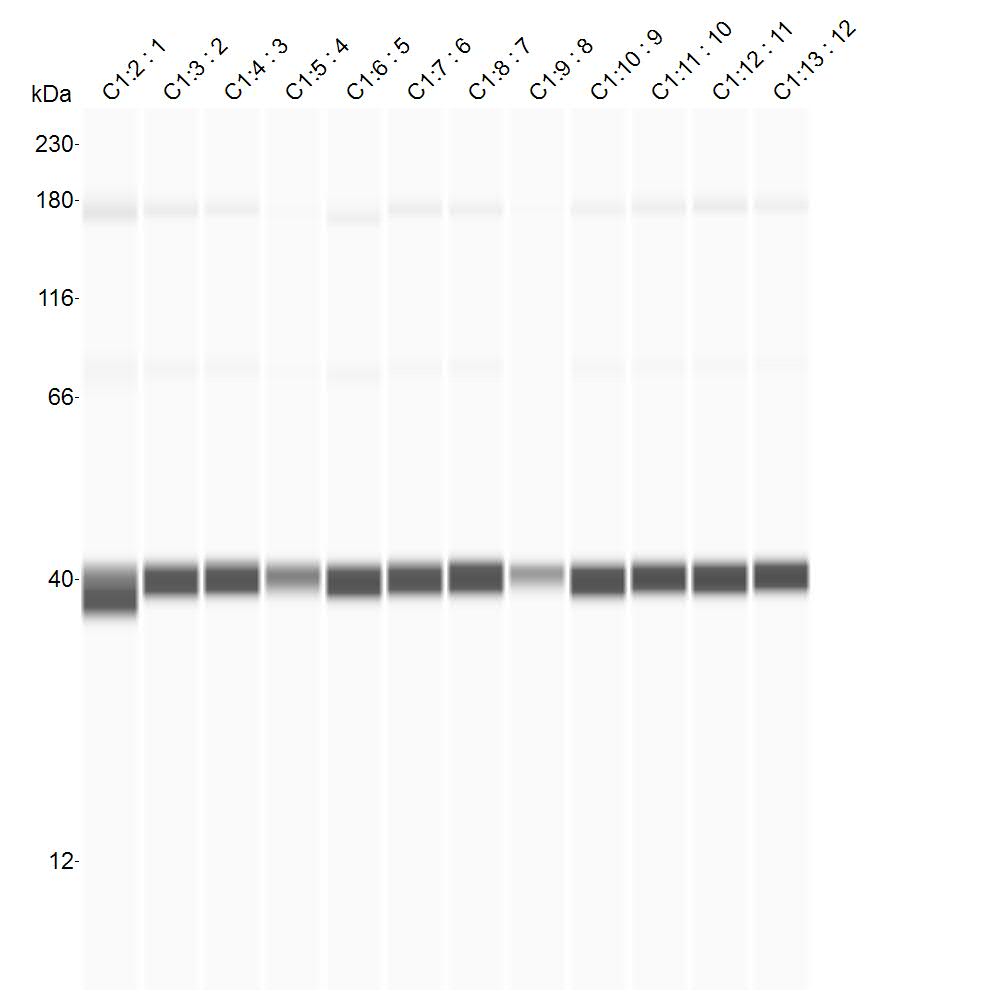

Supplement: S2 Fig — (JPG) [file pone.0242789.s002.jpg]

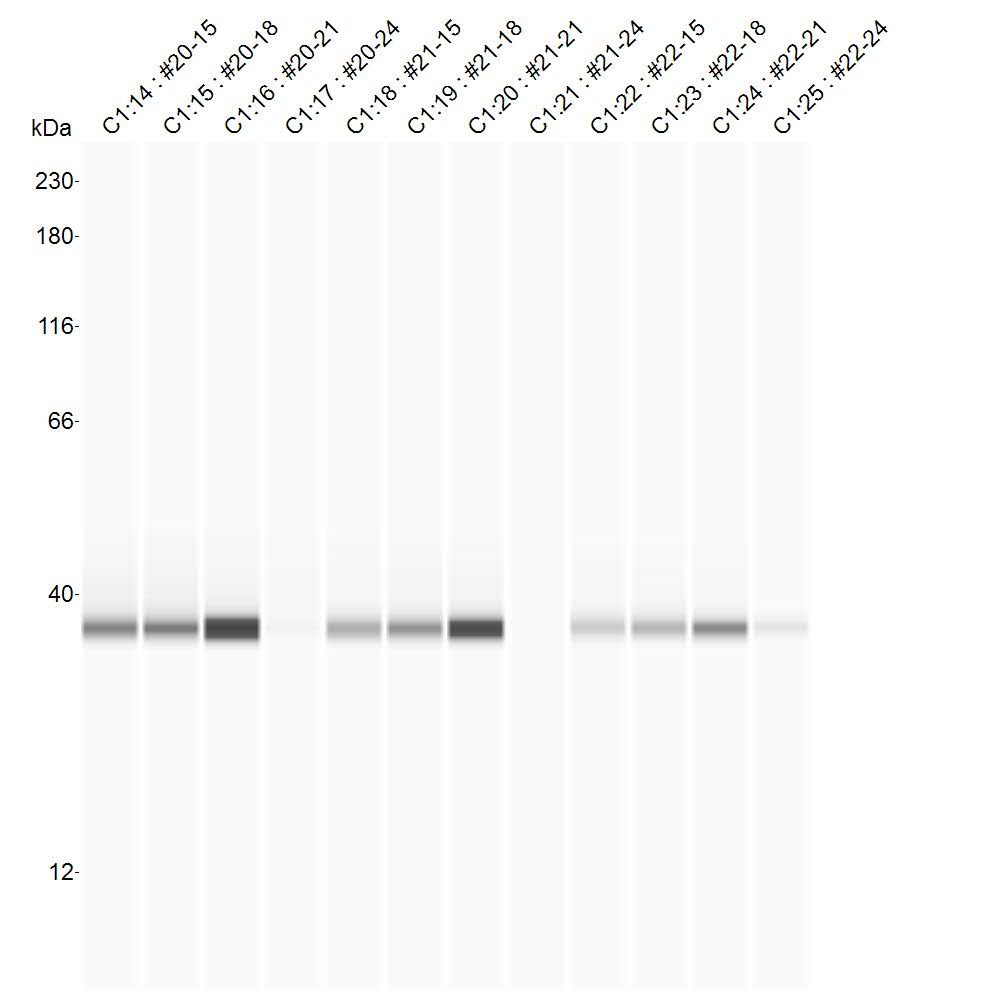

Supplement: S3 Fig — (JPG) [file pone.0242789.s003.jpg]

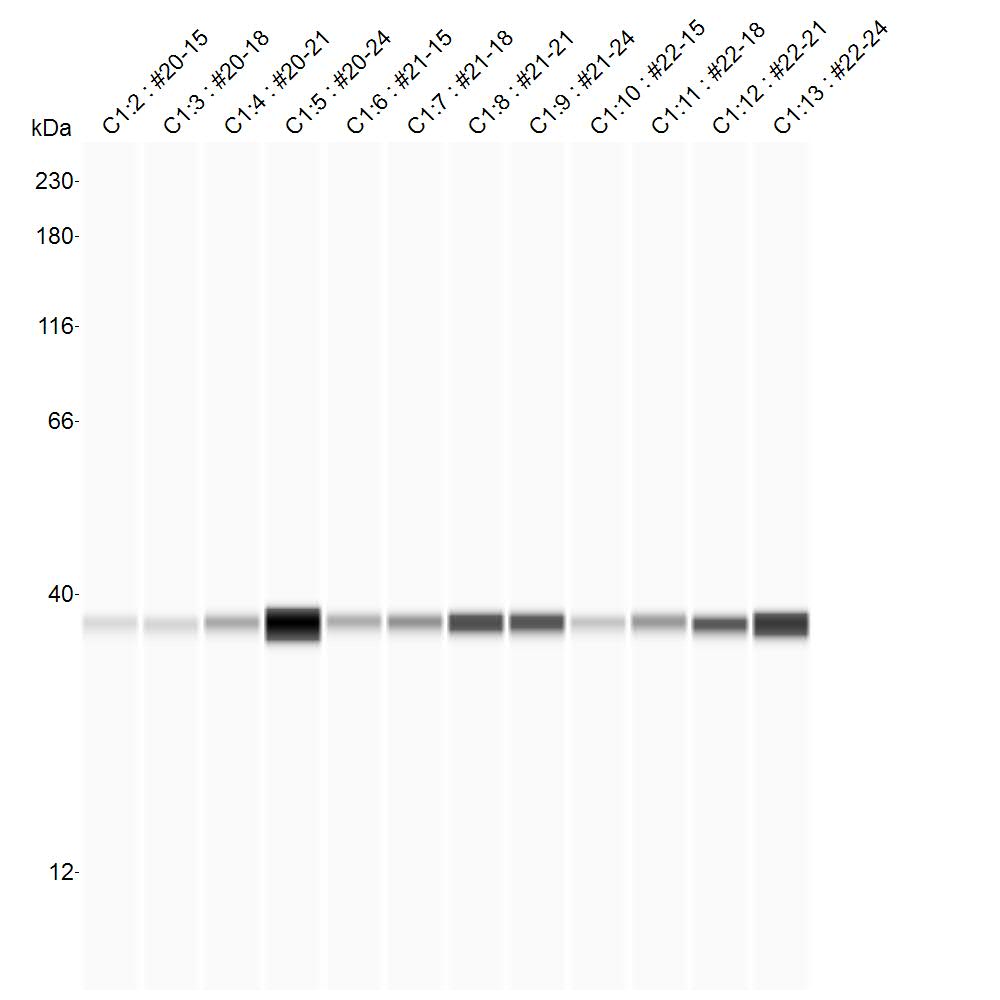

Supplement: S4 Fig — (JPG) [file pone.0242789.s004.jpg]

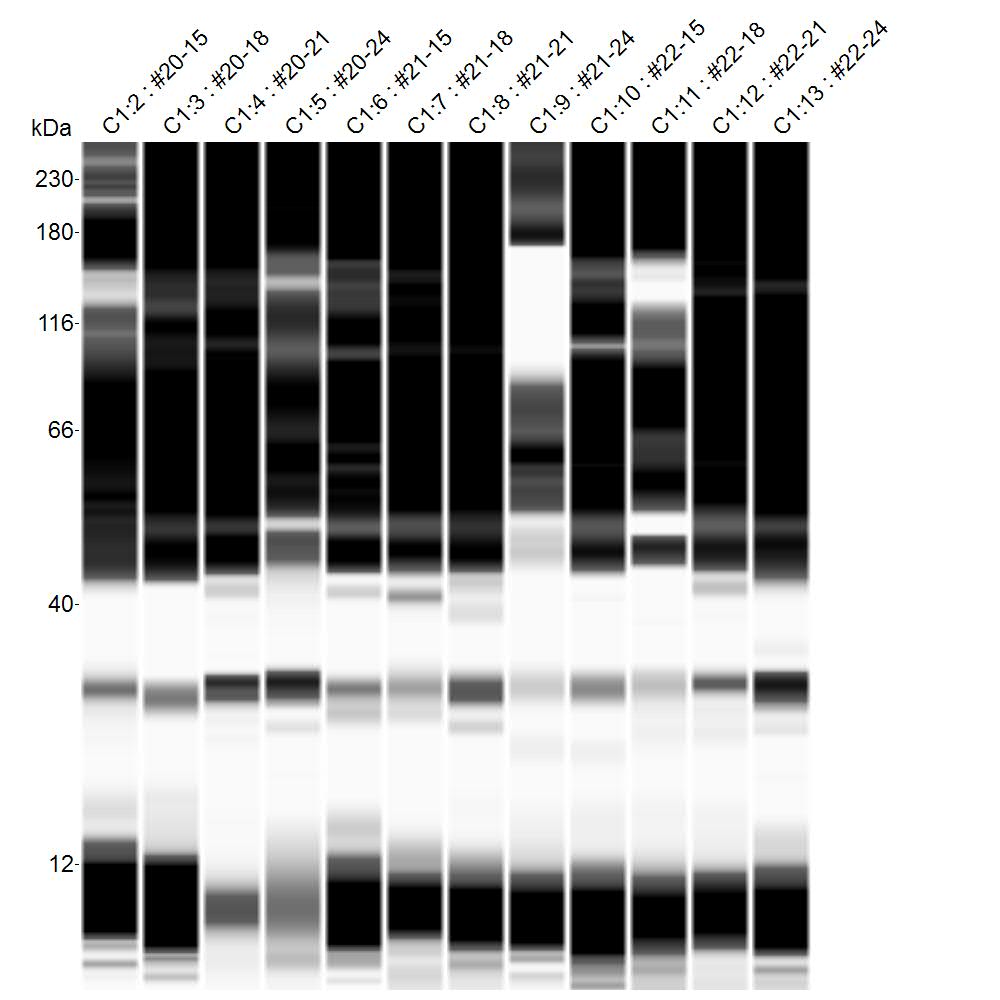

Supplement: S5 Fig — (JPG) [file pone.0242789.s005.jpg]

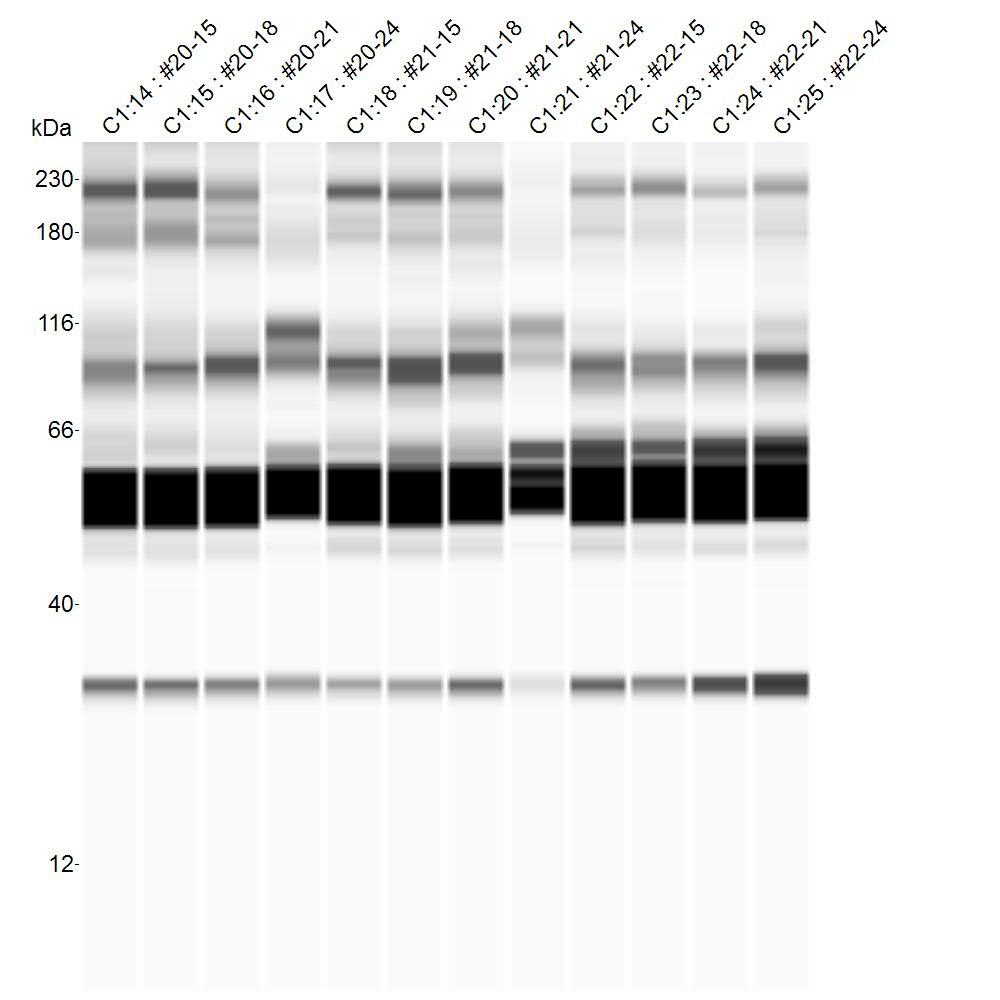

Supplement: S6 Fig — (JPG) [file pone.0242789.s006.jpg]
